# Dimensionality and reliability of the Depression Anxiety Stress Scales 21 among adolescents in North Macedonia

**DOI:** 10.3389/fpsyg.2022.1007594

**Published:** 2022-09-30

**Authors:** Katerina Naumova

**Affiliations:** Department of Psychology, Ss. Cyril and Methodius University in Skopje, Skopje, North Macedonia

**Keywords:** depression, anxiety, stress, DASS-21, adolescence

## Abstract

This study examined the structural validity and reliability of the DASS-21 in a large sample (*N* = 4,202) of secondary school students from North Macedonia (*M_age_* = 16.43 ± 1.04, 65% girls). Based on theoretical and empirical considerations, five structural models were compared using confirmatory factor analysis. The original three-factor model provided good fit to the data; however, high interfactor correlations indicated that the depression, anxiety, and stress factors were indistinguishable. The bifactor solution yielded superior fit relative to other tested models. Factor loading patterns revealed a strong general factor and some specificity of the depression and anxiety factors, whereas the stress items were primarily markers of general distress. Model-based reliability and ancillary bifactor indices revealed that the DASS-21 is essentially unidimensional. Thus, only the total score could be used as a reliable measure of general emotional distress, while subscale scores should be avoided. Overall, the findings provide further support for the cross-cultural validity of the DASS-21 and confirm that it is suitable for use among older adolescents in North Macedonia.

## Introduction

The short form of the Depression Anxiety Stress Scales (DASS-21; Lovibond and Lovibond, 1995) has been widely used to measure emotional distress among adolescents. However, only several studies have examined its factor structure in different cultures using confirmatory factor analysis. Most factor-analytic studies have been conducted with Australian adolescents (Duffy et al., 2005; Tully et al., 2009; Szabó, 2010; Shaw et al., 2017), followed by studies with adolescent samples from Asia (Mellor et al., 2015; Le et al., 2017), South America (Mellor et al., 2015; Patias et al., 2016), Europe (Willemsen et al., 2011; Jovanović et al., 2021) and the United States (Moore et al., 2017). Competing models have emerged in the literature, but the evidence is yet inconclusive whether a bifactor solution is superior to the original three-factor structure or other proposed models.

When Lovibond and Lovibond (1995) initially approached the development of anxiety and depression scales, their intention was to cover the full range of core symptoms while at the same time providing maximum discrimination between the two scales. The factors were defined through clinical consensus, not diagnostic criteria, followed by empirical refinement. The third factor, originally labeled tension/stress, emerged during the analysis of non-discriminating anxiety and depression items, which led to the development of a separate stress scale. The short form of the DASS contains items with the highest factor loadings selected as markers of characteristics specific to each syndrome. Thus, in both versions, the Depression scale assesses anhedonia, dysphoria, hopelessness, devaluation of life, self-deprecation, lack of interest/involvement, and inertia. The Anxiety scale assesses autonomic arousal, skeletal musculature effects, situational anxiety, and subjective experience of anxious affect. Finally, the Stress scale assesses nervous arousal, difficulty relaxing, agitation, irritability, and impatience. Lovibond and Lovibond interpreted the identified moderate intercorrelations between the scales as indicative of a common cause influencing the three states. They have also differentiated the structure of the DASS from the tripartite model of anxiety and depression proposed by Clark and Watson (1991), consisting of general negative affect (NA), physiological hyperarousal (PH; specific to anxiety), and anhedonia/lack of positive affect (PA; specific to depression). The main difference relates to the stress symptoms, interpreted as indicators of general distress in the tripartite model, whereas they define a coherent syndrome within the DASS structure (Lovibond, 1998). Nevertheless, the DASS has been frequently used or assessed as a measure of negative affect based on the tripartite model.

Considering that the DASS was constructed to measure negative affective syndromes present in general and clinical adult populations, research on its utility with adolescents has focused on the incongruity in the structure of these emotional states as experienced by adults and young people, particularly in light of their gradual emergence and differentiation during adolescence. Moreover, although the co-occurrence of depression and anxiety is frequent in adolescents, there is abundant evidence of meaningful distinctions between them (Anderson and Hope, 2008; Cummings et al., 2014). In addition, due to cultural variations in the expression of different modalities of distress among adolescents (Zahn Waxler et al., 2000), assessing the cross-cultural validity of the measure has lately provoked more prominent research interest.

The initial psychometric evaluation of the DASS-21 conducted by Duffy et al. (2005) with younger adolescents (aged 11–15 years) did not find support for the original three-factor model. It also revealed that the three factors were not empirically distinguishable (intercorrelations ≥ 0.90), a finding that has been consistently reported in subsequent studies. Follow-up CFAs on the same data found insufficient fit of the tripartite model of negative affect that led to several model improvements and an acceptable fit of a two-factor solution comprised of generalized negative mood and physiological arousal.

Contrary to this, in a larger young adolescent sample, Szabó (2010) reported acceptable fit of the original model, even though the three factors were highly correlated (*rs* = 0.81–0.93). Nevertheless, the tests of seven additional models revealed that the bifactor model was superior to all alternative models, whereas the unidimensional model had the poorest fit. An alternative bifactor model was also included in these analyses, where the stress items were allocated to load only on the general negative affect factor; however, this model demonstrated inferior fit to the proper bifactor model. Based on the size of factor loadings, Szabo concluded that core symptoms of depression in young adolescents are quite similar to ones previously identified in adults, while the anxiety and stress constructs were less distinct.

In an online study with a representative sample, Tully et al. (2009) found acceptable fit of the original three-factor model in younger but not in older adolescents (15–18 years). The one-dimensional model had poor fit in both age-groups. In contrast, adjustments to a model based on the tripartite conceptualization of NA led to a reasonably good fit of an unrestricted version (Reise et al., 2010; Hyland, 2015) of the alternative bifactor model proposed by Szabo (the group factors of depression and anxiety were allowed to correlate in this analysis). The authors interpreted the findings as evidence of the tripartite structure of emotion, where general NA is synonymous with stress in adolescence. Willemsen et al. (2011) also found support for the alternative bifactor model. Although the original model performed almost as well in both girls and boys in their study, the correlations between the factors were again very high (*rs* = 0.78–0.93). Based on the pattern of factor loadings, the authors concluded that the general factor is more important than the unique factors of depression and anxiety.

Two additional studies have provided support for the original three-factor model, even though they did not test a bifactor solution as well. Mellor et al. (2015) found good fit and factorial invariance of the original model among adolescents from western and eastern cultures, while Patias et al. (2016) demonstrated superior fit of the original model relative to the one-dimensional model and a two-factor model with the anxiety and stress items loading on one factor.

On the other hand, studies that have employed significantly larger samples (1,300–3,000 students) have shown that the restricted (Reise et al., 2010) bifactor solution outperformed other models. Cross-cultural evidence on the superiority of the bifactor structure of the DASS-21 among adolescents has recently been mounting. For example, Shaw et al. (2017) found excellent fit of the bifactor model in both younger and older girls and boys. They also found good fit of the original model, albeit with very high interfactor correlations (*rs* = 0.85–0.97). Additional support for the relevance of an underlying general psychological distress factor was provided by the very high values of the omega hierarchical coefficients for the total score (ωh = 0.92–0.95) relative to omega hierarchical subscale values (ωhs = 0.04–0.20). Likewise, in a sample of older adolescents, Moore et al. (2017) found that the bifactor solution was superior to the three-dimensional, one-dimensional and alternative bifactor models. They reported negligible omega hierarchical subscale values (ωhs ≤ 0.03), indicating that the majority of variance was explained by general negative affectivity, implying that subscale scores were unreliable.

Le et al. (2017) further corroborated these findings after testing several models and showing acceptable fit of a correlated bifactor model with three specific factors, as opposed to poor fit of the one-dimensional, three-dimensional, and other hypothesized and empirically modified models. Finally, in an attempt to surpass the limitations of traditional CFA models, Jovanović et al. (2021) employed exploratory structural equation modeling (ESEM) to compare seven structural models using CFA with two additional models using ESEM. Again, the one-dimensional model had the poorest, although acceptable, fit. The three-factor model had good fit, while the bifactor-ESEM model had the best fit, followed by the bifactor CFA and ESEM models. They also found that the depression and anxiety subscales had certain amount of specificity over and above the general factor (ωh = 0.89 vs. ωhs of 0.20 and 0.16, respectively).

It is of note that apart from inconsistent findings relating to the internal validity of the DASS-21, there have been inconsistencies across studies relating to the terminology and employed CFA parameter estimators. For example, the bifactor model has occasionally been labeled quadripartite (Szabó, 2010), hierarchical (Willemsen et al., 2011), or a four-factor model (Le et al., 2017). Furthermore, due to the ordinal response scale of the DASS, most studies have used the WLSMV or other robust estimator; however, few have employed ML (Duffy et al., 2005; Szabó, 2010; Le et al., 2017). Additionally, empirical modifications to examined models have increased the number of hypothesized structural models in the literature while at the same time decreasing the possibilities of their reasonable interpretation (Markon, 2019).

Given the reported variations in factor structure across samples, the aims of this study were twofold. First, to test several competing models of the DASS-21 among older adolescents in the Republic of North Macedonia, and second, to examine the reliability of the measure.

## Materials and methods

### Participants and procedure

The sample consisted of 4,202 students (64.9% girls) from public secondary schools in 25 municipalities from all regions of the Republic of North Macedonia between the ages of 15 and 18 (M = 16.43, SD = 1.04). Participants were primarily in their second year of study (31.2%), followed by first year (28.8%), third year (26.5%), and fourth year (13.5%). Significant gender differences regarding year of study were not found [*χ*^2^(3) = 2.51, *p* = 0.473].

The data used in this research were collected as part of a larger project on the measurement of adolescent risk behavior funded by the Faculty of Philosophy at the Ss. Cyril and Methodius University in Skopje. In accordance with local legislation, approvals to conduct the study were obtained from the Ministry of Education and Science of North Macedonia and from the principals of the schools invited to participate. Emails with a link to the survey hosted on Qualtrics were distributed to students by school staff in March 2021. The link was active for 2 weeks. Students were informed of the voluntary nature of their participation in the study and the anonymity of data. Consent to participate was implied by the completion of the survey.

### Instrument

An existing unpublished translation of the DASS in Macedonian language was used that incorporates linguistic and cultural adaptations emphasizing conceptual rather than literal similarity of items (van de Vijver, 2016). Each of the three scales comprising the short form of the measure consists of 7 items describing various expressions of negative emotional states. Participants rate the severity/frequency of symptoms experienced in the past week on a 4-point scale (from 0 = *did not apply to me at all* to 3 = *applied to me very much or most of the time*). The scores range from 0 to 21 on each subscale and from 0 to 63 on the total scale. In the present sample, the following Cronbach’s alpha coefficients were obtained: 0.89 for Depression, 0.85 for Anxiety, 0.84 for Stress, and 0.94 for the total scale.

### Statistical analysis

Statistical analyses were conducted using SPSS 24.0, lavaan (Rosseel, 2012), and semtools (Jorgensen et al., 2022) packages for R (R Core Team, 2022), and Omega software (Watkins, 2013). For the CFA, the weighted least squares with mean and variance adjusted (WLSMV) estimator was used due to the four-point response scale. Even though cutoff criteria for WLSMV fit indices have not been suggested in the literature, the above-cited studies using the same estimator have referred to recommendations by Hu and Bentler (1999); thus they were also employed in this study to evaluate overall model fit (CFI ≥ 0.95, RMSEA ≤ 0.06; SRMR ≤ 0.08).

**Figure 1:**
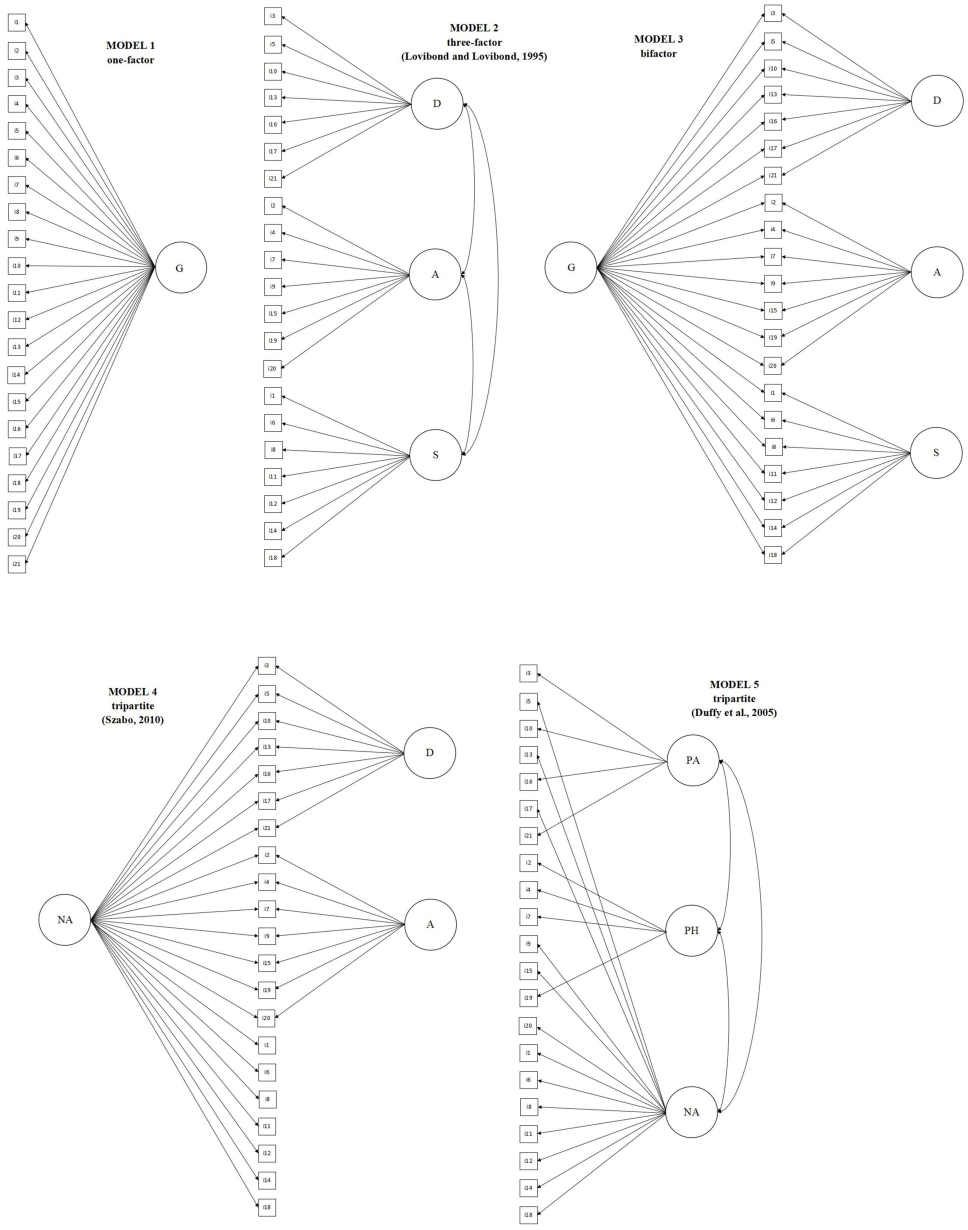
Five alternative models of the DASS-21 evaluated in the study.

Five models were tested (Figure 1): (a) Model 1: a one-dimensional model; (b) Model 2: the original three-factor model (Lovibond and Lovibond, 1995); (c) Model 3: a bifactor model with one general factor and three specific factors (depression, anxiety, and stress); (d) Model 4: a tripartite model consisting of a general NA factor on which stress items are allocated to load and two specific factors (depression and anxiety; Szabó, 2010); (e) Model 5: an alternative tripartite model comprised of three factors, physiological arousal (items 2, 4, 7, and 19), lack of positive affect (items 3, 10, 16, and 21) and generalized negativity (the remaining 13 items; Duffy et al., 2005). The models were tested without empirical modifications.

Omega indices were used to estimate model-based reliability of the DASS-21. Omega total (ω/ωs) estimates the proportion of total/subscale score variance that can be explained by common variance in the model, while omega hierarchical (ωh/ωhs) estimates the proportion of total/subscale score variance explained by the general, i.e., specific factors within a bifactor model. Relative omega indicates the proportion of reliable variance (PRV) in the multidimensional composite due to the general factor, i.e., in the subscale composite independent of the general factor.

Following recommendations by Rodriguez et al. (2016), additional statistical indices were calculated to assist the understanding of the dimensionality of data. Explained common variance (ECV) represents the proportion of common variance explained by the general factor and is therefore defined as a true unidimensionality index. It is moderated by the percent of uncontaminated correlations (PUC), which estimates the proportion of correlations between items not contaminated by multidimensionality. The bias of forcing multidimensional data into a unidimensional model is estimated by the average relative parameter bias (ARPB), i.e., the average difference between factor loadings on the general factor in a bifactor model and the factor loadings in a unidimensional model. Finally, the factor determinacy index (FDI) assesses how well factor scores estimate an underlying latent variable, whereas construct reliability/replicability (H) assesses to what extent a set of items represents a latent variable adequately.

## Results

Descriptive statistics for the DASS-21 subscales and total scale are presented in Table 1. Gender differences are presented in Table 2 and reveal that girls reported more symptoms of depression, anxiety, and stress. Significant age differences were not observed.

**Table 1:** Descriptive statistics for the DASS-21.

Scale	M	SD	Skewness	Kurtosis
Depression	3.98	4.51	1.49	1.86
Anxiety	4.28	4.34	1.36	1.48
Stress	6.08	4.59	0.81	0.10
Total	14.34	12.21	1.18	0.98

**Table 2:** Mean differences between girls and boys.

DASS-21	Girls (*n* = 2,725)		Boys (*n* = 1,477)		Welch’s *t*	Cohen’s *d*
	M	SD	M	SD		
Depression	4.59	4.67	2.85	3.96	12.76^***^	0.40
Anxiety	5.19	4.64	2.59	3.08	21.78^***^	0.66
Stress	6.97	4.69	4.44	3.90	18.70^***^	0.59
Total	16.76	12.71	9.88	9.78	19.54^***^	0.61

*** *p* < 0.001.

The goodness-of-fit indices of the five competing models of the DASS-21 are presented in Table 3. The unidimensional model (Model 1) had the poorest fit relative to other models (*χ*^2^ = 5130.66, *p* < 0.001; CFI = 0.95, RMSEA = 0.08, SRMR = 0.05). The correlated three-factor model (Model 2) proposed by Lovibond and Lovibond (1995) fitted the data well, although the RMSEA value was marginal (*χ*^2^ = 2954.12, *p* < 0.001; CFI = 0.97, RMSEA = 0.06, SRMR = 0.04). However, the correlations among the three factors were very high (D-A *r* = 0.82, D-S *r* = 0.90, A-S *r* = 0.91), indicating that the factors are not distinguishable. The bifactor model with one general and three specific factors (Model 3) achieved the best fit (*χ*^2^ = 1469.83, *p* < 0.001; CFI = 0.99, RMSEA = 0.04, SRMR = 0.03). From the remaining two models based on the tripartite conceptualization of depression and anxiety, the alternative bifactor model (Model 4) initially proposed by Szabó (2010) had a better fit (*χ*^2^ = 2329.40, *p* < 0.001; CFI = 0.98, RMSEA = 0.05, SRMR = 0.03) than the three-factor model (Model 5) proposed by Duffy et al. (2005) (*χ*^2^ = 4022.45, *p* < 0.001; CFI = 0.96, RMSEA = 0.07, SRMR = 0.04). Furthermore, the interfactor correlations in this model were also high (PH-PA *r* = 0.71, PH-NA *r* = 0.88, PA-NA *r* = 0.91). Although both bifactor models fitted the data well, based on statistical and conceptual considerations, the analysis further focused on Model 3, given that it can provide factor-level and item-level information on the stress factor that the authors of the DASS-21 have defined as a separate syndrome (Lovibond, 1998).

**Table 3:** Fit indices of the evaluated CFA models.

Model	WLSMV *χ*^2^	*df*	CFI	RMSEA [90%CI]	SRMR
1. One-factor	5130.66^***^	189	0.946	0.079 [0.077, 0.081]	0.053
2. Three-factor	2954.12^***^	186	0.970	0.060 [0.058, 0.061]	0.038
3. Bifactor	1469.83^***^	168	0.986	0.043 [0.041, 0.045]	0.026
4. Tripartite^a^	2329.40^***^	175	0.976	0.054 [0.052, 0.056]	0.032
5. Tripartite^b^	4022.45^***^	186	0.958	0.070 [0.068, 0.072]	0.044

a Bifactor model with one general factor on which stress items are allocated to load and two specific factors of depression and anxiety (Szabó, 2010).

b Three-factor model comprised of physiological arousal, lack of positive affect, and generalized negativity (Duffy et al., 2005).

*** *p* < 0.001.

Standardized factor loadings for the bifactor model are presented in Table 4. All DASS-21 items had significant and high loadings on the general factor (range = 0.509–0.896, mean = 0.701). All depression, anxiety, and stress items also had significant loadings on their specific factors, although substantially smaller than on the general factor (mean = 0.363, mean = 0.343, and mean = 0.151, respectively). Items 5 and 13 (indicators of inertia and dysphoria) had the smallest loadings on the depression factor (0.267 and 0.209, respectively). Furthermore, anxiety item 2 (“mouth dryness”) and item 9 (“worry about panic”) had very low loadings (0.112 and 0.158, respectively) on the specific factor. Five stress items also had small loadings on the stress factor, while three of those were negative (items 1, 11, and 12—indicators of difficulty relaxing and agitation). In contrast, item 6 (“tend to overreact”) had an equally high loading on both the general (0.627) and the stress factor (0.612). Item-level explained common variance provided further support that most stress items are pure markers of general emotional distress (I-ECV > 0.85; Rodriguez et al., 2016), while the depression and anxiety factors have some real meaning.

**Table 4:** Standardized factor loadings for the bifactor model of the DASS-21.

Item	General	Depression	Anxiety	Stress	I-ECVI
3	0.634^***^	0.374^***^			0.742
5	0.738^***^	0.267^***^			0.884
10	0.707^***^	0.450^***^			0.712
13	0.852^***^	0.209^***^			0.943
16	0.653^***^	0.408^***^			0.719
17	0.721^***^	0.402^***^			0.763
21	0.737^***^	0.433^***^			0.743
2	0.509^***^		0.112^***^		0.954
4	0.616^***^		0.464^***^		0.638
7	0.700^***^		0.367^***^		0.784
9	0.791^***^		0.158^***^		0.962
15	0.804^***^		0.326^***^		0.859
19	0.564^***^		0.459^***^		0.602
20	0.743^***^		0.285^***^		0.872
1	0.742^***^			−0.113^***^	0.977
6	0.627^***^			0.612^***^	0.512
8	0.705^***^			0.352^***^	0.800
11	0.896^***^			−0.053^**^	0.997
12	0.764^***^			−0.046^*^	0.996
14	0.581^***^			0.136^***^	0.948
18	0.629^***^			0.170^***^	0.932

I-ECV, item explained common variance.

* *p* < 0.05;

** *p* < 0.01 and

*** *p* < 0.001.

As shown in Table 5, the general factor explained 50% of the total variance and 82% of the common variance, whereas the specific factors explained 2.7%–4.6% of the total variance and 4.4%–7.6% of the common variance. Considering that the explained common variance value (ECV) for the general factor exceeded the recommended benchmark of 0.70 (Rodriguez et al., 2016), whereas the percent of uncontaminated correlations did not (PUC = 0.70), the relative parameter bias was also calculated. The average bias across items (ARPB) was 4.4%, implying that the data are essentially unidimensional (Reise et al., 2013).

**Table 5:** Omega reliability and ancillary indices for the bifactor model.

Indices	General	Depression	Anxiety	Stress
Total variance	0.500	0.046	0.037	0.027
Common variance (ECV)	0.819	0.076	0.061	0.044
Omega	0.965	0.931	0.902	0.899
Omega (h)	0.914	0.189	0.157	0.039
Relative omega (PRV)	0.946	0.203	0.174	0.044
H	0.962	0.538	0.485	0.447
FDI	0.981	0.733	0.697	0.668

ECV, explained common variance; PRV, proportion of reliable variance; H, index of construct replicability; FDI, factor determinacy index.

The finding that most of the variance in the DASS-21 is explained by the general factor, even though the Depression and Anxiety subscales have some specificity over and above the general factor relative to the Stress subscale, was further evidenced by the omega reliability indices. The coefficients of composite reliability exceeded the recommended benchmark of 0.80 (Reise et al., 2013), both for the total score (ω = 0.96) and the subscales (ωs = 0.90–0.93); however, omega hierarchical coefficients confirmed that the general factor explained most of the variance of the total score and the subscale scores. Relative omega coefficients for the subscales (ωhs/ωs) also indicated that after controlling for the general factor, the specific factors of depression and anxiety provide some unique information (20% and 17%, respectively), while the stress factor does not (4%).

Factor determinacy values for the group factors were well below the recommended benchmark of 0.90 (Rodriguez et al., 2016), implying that only the factor scores from the general factor are trustworthy (FDI = 0.98). The construct replicability indices of the group factors were also below the suggested criterion of H = 0.70 (Rodriguez et al., 2016), indicating that the factors are not defined well by their indicators and are expected to change across studies. In contrast, the general factor is represented very well (H = 0.96) and is expected to be stable across studies.

## Discussion

This study examined the dimensionality and model-based reliability of the DASS-21 in older adolescents from North Macedonia. Five alternative models were compared using CFA based on theoretical and empirical considerations. As previously evidenced, the unidimensional model had the poorest fit. The correlated three-factor model initially proposed by Lovibond and Lovibond (1995) yielded good fit; however, consistent with previous studies (Tully et al., 2009; Willemsen et al., 2011; Moore et al., 2017; Shaw et al., 2017), high interfactor correlations indicated that the depression, anxiety, and stress factors were empirically indistinguishable. The two models based on the tripartite conceptualization of anxiety and depression (Clark and Watson, 1991) performed disparately. The model proposed by Duffy et al. (2005), consisting of physiological arousal, low positive affect, and generalized negativity, had an acceptable but poorer fit than the original three-factor model, similar to which it could not distinguish the three proposed factors empirically. The tripartite bifactor model proposed by Szabó (2010), comprised of a general negative affect factor and two specific depression and anxiety factors while treating the stress items only as indicators of general distress, provided good fit; however, the bifactor model with three specific factors was selected as more meaningful for further analysis, based on the goodness-of-fit indices and the comprehensiveness of information it provided.

The examination of factor loading patterns for this bifactor model revealed a strong general factor and some specificity of the Depression and Anxiety factors. In particular, all items loaded more strongly on the general factor than the specific factors, except one item from the stress subscale (item 6, “tendency to overreact”). Nevertheless, most items on the Depression and Anxiety factors had acceptable loadings. Only the indicators of dysphoria and inertia had a weak relationship with the Depression factor, whereas the indicator of situational anxiety and one indicator of autonomic arousal (“dry mouth”) had small loadings on the Anxiety factor. Consistent with previous studies, the Depression factor had higher specificity than the Anxiety factor, while the Stress factor was least distinguishable (Moore et al., 2017; Shaw et al., 2017; Jovanović et al., 2021), with most items loading highly on the general factor and very weakly on the specific factor. Item-level explained variance further corroborated these findings.

Dimensionality and model-based reliability indices indicated that the DASS-21 could be modeled and scored as essentially unidimensional. Omega hierarchical coefficients showed that the general factor explained the majority of variance of the total score and the subscale scores, even though the Depression and Anxiety factors provide some unique information, whereas the Stress factor does not. The construct replicability index and factor determinacy index further revealed that only the general factor is defined adequately by its indicators and is expected to be stable across studies; thus, only its factor scores are trustworthy.^1^

The findings are in line with previous cautionary suggestions that only the total score of the DASS-21 could be used as a reliable indicator of general emotional distress in adolescents (Le et al., 2017; Moore et al., 2017; Shaw et al., 2017; Jovanović et al., 2021). Even though the Depression and Anxiety factors provide some specificity over and above the general factor, subscale scores are not reliable, and their use should be avoided. The Stress subscale is least advisable to be used as a unique indicator of stress-related symptoms. Considering the age range of the sample, the findings further indicate that these negative emotional states are not differentiated well in middle to late adolescence.

Several issues raised since the onset of confirmatory factor-analytic studies with adolescent samples have also been evidenced in this study. Namely, whether stress, as conceptualized by Lovibond and Lovibond (1995), differentiates from general distress later in the development continuum or a new conceptualization of stress is needed that captures the specificity and variability of this state during adolescence. Additionally, mouth dryness, as well as worry about panic, might not be strong indicators of autonomic arousal in adolescents (Le et al., 2017; Moore et al., 2017; Shaw et al., 2017; Jovanović et al., 2021). Similar findings have been reported regarding the indicators of inertia and dysphoria (Le et al., 2017; Shaw et al., 2017; Jovanović et al., 2021), which were the weakest markers of depression in this sample. Possible solutions include omitting, revising, or replacing some or all of these items. A recently published revision of the DASS intended for youth (DASS-Y; Szabo and Lovibond, 2022) provides initial data on the utility of simplifying and clarifying item wordings while retaining indicators of all specific aspects of each emotional state, except inertia.

In conclusion, the study provided further evidence of the cross-cultural validity of the DASS-21 when utilized with adolescents. Even though the sample was large and heterogeneous, it should be noted that girls were slightly overrepresented, and the measure was administered online during the Covid-19 pandemic; however, the obtained scores did not differ substantially from previously reported data. Overall, the findings indicate that the DASS-21 could be used as a brief and psychometrically sound measure of general emotional distress in research and community contexts. The ancillary bifactor indices ambiguously verified the unidimensionality of the measure, despite the inherent multidimensionality of the data. Due to a lack of validated measures in the Macedonian language, the construct validity could not be adequately investigated; however, future studies should examine the factor structure of the DASS-21 in clinical samples of adolescents and its gender invariance. In addition, longitudinal research could provide insight into the trajectories of anxiety and depression during adolescence and help disentangle their distinctive and overlapping features.

## Data availability statement

The raw data supporting the conclusions of this article will be made available by the authors, without undue reservation.

## Ethics statement

In accordance with local legislation, approvals to conduct the study were obtained from the Ministry of Education and Science of North Macedonia and from the principals of the schools invited to participate. Students were informed of the voluntary nature of their participation in the study and the anonymity of data. Consent to participate was implied by the completion of the survey.

## Author contributions

The author confirms being the sole contributor of this work and has approved it for publication.

## Conflict of interest

The author declares that the research was conducted in the absence of any commercial or financial relationships that could be construed as a potential conflict of interest.

## Publisher’s note

All claims expressed in this article are solely those of the authors and do not necessarily represent those of their affiliated organizations, or those of the publisher, the editors and the reviewers. Any product that may be evaluated in this article, or claim that may be made by its manufacturer, is not guaranteed or endorsed by the publisher.

## Footnotes

^1^Ancillary indices for the bifactor model proposed by Szabó (2010) were calculated as well, and they did not lend support for such tripartite modeling of DASS-21 data. This model was also essentially unidimensional.
